# Calcipotriol Inhibits NLRP3 Signal Through YAP1 Activation to Alleviate Cholestatic Liver Injury and Fibrosis

**DOI:** 10.3389/fphar.2020.00200

**Published:** 2020-03-31

**Authors:** Xiaopeng Wang, Guiyang Wang, Junwen Qu, Zhiqing Yuan, Ruogu Pan, Kewei Li

**Affiliations:** ^1^Department of Biliary-Pancreatic Surgery, Renji Hospital, School of Medicine, Shanghai Jiao Tong University, Shanghai, China; ^2^The First Department of Hepatic Surgery, Eastern Hepatobiliary Surgery Hospital, Naval Medical University, Second Military Medical University, Shanghai, China

**Keywords:** cholestasis, fibrogenesis, calcipotriol, NLRP3 inflammasome, VDR, YAP1

## Abstract

Cholestasis is common in multiple clinical circumstances. The NOD-like receptor protein 3 (NLRP3) inflammasome pathway has been demonstrated to play an important role in liver injury and fibrosis induced by cholestasis. We previously proved that MCC950, a selective NLRP3 inhibitor, alleviates liver fibrosis and injury in experimental liver cholestasis induced by bile-duct ligation (BDL) in mice. Herein, we investigate the role of calcipotriol, a potent vitamin D receptor agonist, in experimental liver cholestasis, test its therapeutic efficacy, and explore its potential protective mechanism. C57BL/6 mice were made to undergo BDL or fed the 0.1% 3,5-diethoxycarbonyl-1,4-dihydrocollidine (DDC) diet to establish two classic cholestatic models. Calcipotriol was administered intraperitoneally to these mice daily. Serum makers of liver damage and integrity, liver histological changes, levels of liver pro-fibrotic markers, bile acid synthetases and transporters were measured *in vivo*. The underlying mechanism by which calcipotriol alleviates cholestatic liver injury and fibrosis was further investigated. The results of the current study demonstrated that calcipotriol supplement significantly alleviate cholestatic liver injury and fibrosis. Moreover, calcipotriol supplement markedly inhibited NLRP3 inflammasome pathway activation to alleviate liver injury and fibrosis *in vivo* and inhibit hepatic stellate cell (HSC) activation *in vitro*. In addition, VDR agonist calcipotriol exert inhibitory effect on NLRP3 inflammasome activation through activating yes-associated protein 1 (YAP1). In conclusion, our findings proved that calcipotriol suppressed the NLRP3 signal by activating YAP1 to alleviate liver injury and retard fibrogenesis in cholestasis.

## Introduction

Cholestasis is a common clinical syndrome of liver dysfunction characterized by impaired bile secrection and flow that can worsen ongoing liver disease. Multiple etiologies can cause cholestasis, such as extrahepatic biliary obstruction due to biliary stones, cholangiocarcinoma, and biliary atresia and intrahepatic cholestasis due to viral hepatitis, autoimmune diseases, and pregnancy (Kullak-Ublick and Meier, 2000; Yu et al., 2017). Without effective treatment, the accumulation of toxic bile acid (BA) can cause liver damage, fibrosis, or even cirrhosis, leading eventually to liver function failure (Li and Apte, 2015; Wu et al., 2017). However, the drugs currently applied to clinical treatment of cholestasis are extremely rare due to either poor efficacy or high cost (Goldstein and Levy, 2018). The treatment for cholestasis is controversial and alternative effective therapy is still urgently needed.

Inflammation and cell death are two vial elements in the development of liver injury and fibrosis in cholestasis. The NOD-like receptor protein 3 (NLRP3), the most well-studied inflammasome, is a multiprotein cytoplasmic complex comprising apoptosis-associated speck-like protein (ASC) and the effector molecule pro-caspase-1 (Ting et al., 2008). Stimulated by pathogen-associated molecular patterns and damage-associated molecular patterns, activated NLRP3 inflammasome governs the cleavage and activation of capase-1, leading to the maturation and extracellular secretion of IL-1β and IL-18, which serve as proinflammatory cytokines in multiple pathophysiological diseases (Dinarello, 2009). Recent studies have highlighted the critical role of NLRP3 in multiple liver disorders and fibrogenesis (Wree et al., 2014; Gong et al., 2016; Mridha et al., 2017). Moreover, activation of the NLRP3 pathway promotes hepatic stellate cell (HSC) activation, which is critical for the initiation and development of liver fibrosis (Watanabe et al., 2009; Inzaugarat et al., 2019). In a previous study, we proved that NLRP3 inhibition by MCC950, a selective NLRP3 inhibitor, can significantly alleviate liver injury and retard the development of fibrosis in bile-duct-ligation (BDL)-induced cholestasis in mice (Qu et al., 2019). Therefore, inhibiting activation of the NLRP3 inflammasome may be a potential therapeutic route for effectively treating cholestatic liver injury and fibrosis.

Vitamin D receptor (VDR), a member of nuclear receptor superfamily, plays an important role in regulating mineral and bone homeostasis. However, because of the weak expression of VDR in hepatic parenchymal cells (Gonzalez-Sanchez et al., 2017), its physiological role in the liver has been ignored since long. According to previous studies, VDR is expressed in most immune cells and plays an important role in modulating both innate and adaptive immune systems. Multiple studies have demonstrated that VDR polymorphisms are associated with the occurrence of a wide range of liver disorders. The research of Christos proved that the *Apa*I, *Taq*I, and *Bsm*I polymorphisms are associated the severity of liver cirrhosis, through the immunoregulatory process (Triantos et al., 2018). Peng et al. (2014) demonstrated that VDR rs2228570 may contributed to increased susceptibility to HBV-related HCC in the Chinese population. Fang et al. (2015) proved a close link between VDR *Bsm*I polymorphism and primary biliary cirrhosis in Asians. However, due to ethnic and geographical differences, the experimental results are somewhat inconsistent and the potential mechanism of the impact of VDR polymorphisms on different disorders still need in-depth researched. Moreover, VDR governs the TGF-β/smad signal, thus playing an important role in modulating fibrosis (Ding et al., 2013; Beilfuss et al., 2015; Duran et al., 2016). In cholestasis, VDR can regulate the metabolism and transport of BA, and VDR deficiency promotes liver injury in mice (Ogura et al., 2009; Ishizawa et al., 2012; Firrincieli et al., 2013). Multiple recent studies highlight anti-inflammatory effect of VDR which is very likely to serves as a potentially effective therapeutic strategy for liver diseases and urgently needs in-depth researched. In previous related studies, Huang et al. demonstrated that VDR could bind NLRP3 to restrict allgergic response via inhibiting IL4 gene transcription and preventing CD4+ T cell Th2 polarition (Huang et al., 2018). Further mechanistic research proved that VDR ligand calcitriol exert inhibiting effect on NLRP3 pathway via Nrf2 activation in human corneal epithelial cells and may serves a potential therapy of dry eye which is a common ocular surface problem (Dai et al., 2019). However, the regulatory effect of VDR ligands on NLRP3 signal and immunomodulatory properties was controversial, the study of Tulk et al. (2015) Proved that 1,25(OH)2D3, a VDR active ligand, could enhance the NLRP3 activation and the secrection of its downstream proinflammatory effector IL-1β in human THP-1 monocytic cells. Given the results of several previous studies, hence, we hypothesize the existence of a close relationship between VDR and the NLRP3 inflammasome signal and assume that NLRP3 inhibition through VDR activation may induce anti-fibrosis and anti-inflammatory effects in cholestatic liver injury, which may serve as a new mechanism of the liver-protective effect of VDR.

In this study, we employ calcipotriol, a potent VDR agonist, to test its therapeutic effect in cholestasis, determine whether VDR can modulate NLRP3 to inhibit HSC activation and alleviate liver fibrosis and injury, and explore the underlying mechanism.

## Materials and Methods

### Reagents and Antibodies

Reagents were purchased as follows: DDC (Sigma-Aldrich, 137030), DMSO (Sigma-Aldrich, 543900), LPS (Sigma-Aldrich, L2630), ATP (YEASEN, 60605ES03), calcipotriol (MCE, HY-10001), calcifediol (MCE, HY-32351), MCC950 (MCE, HY-12815); The antibodies used for western blot were purchased as follows: NLRP3 (Cell Signaling Technology, 15101), caspase-1 p20 (Santa Cruz Biotechnology, sc-398715), ASC (Santa Cruz Biotechnology, sc-514414), IL-1β (Cell Signaling Technology, 12426), α-SMA (ABclonal, A17910), Col1A1 (ABclonal, A1352), β-actin (ABclonal, AC026), YAP1 (proteintech, 13584-1-AP), p-YAP1 (Cell Signaling Technology, 13008), VDR (ABclonal, A2194), CYP7A1 (ABclonal, A10615), MRP3 (Cell Signaling Technology, 39909), CYP8B1 (Abcam, ab191910). The antibodies used for IHC were purchased as follows: NLRP3 (ABclonal, A12694), α-SMA (ABclonal, A17910), Col1A1 (ABclonal, A1352).

### Animals

Male, 8-week-old C57BL/6 mice with an average weight of 19–22 g were used for all experiments. Mice were maintained in a specific pathogen free animal facility under a 12-h daylight, 12-h night cycle. Calcipotriol (MedChemExpress, China) was dissolved in DMSO (Sigma-Aldrich, United States) and then diluted with saline. Saline with the same concentration of DMSO was employed as vehicle.

#### Bile Duct Ligation (BDL)-Induced Cholestasis

Bile duct ligation surgeries were performed as previous described (Qu et al., 2019) and sham operation group underwent the same procedure except for BDL and served as healthy controls. The low dose (LD; 80 μg/kg) of calcipotriol were referenced from a previous study and proved to be most effective in anti-fibrosis and liver protection in that *in vivo* experiment (Wahsh et al., 2016). We take an unstudied dose as the high dose (HD; 160 μg/kg) to explore it therapeutic efficacy in BDL-induced cholestasis. Calcipotriol was administered intraperitoneally daily. Mice were randomly assigned to groups (*n* = 10 per group) as follows: sham operation + vehicle group (sham), sham operation + calcipotriol group (Cal), BDL + vehicle group (BDL), BDL + low dose calcipotriol group (BDL + LD Cal) and BDL + high dose calcipotriol group (BDL + HD Cal). The mice were sacrificed after anesthesia 7 days after surgery. The livers, kidneys and serum were harvested and stored at −80°C.

#### DDC-Induced Cholestasis

Cholestatic liver injury was also induced with a 0.1%DDC-containing diet as described (Lua et al., 2016) and mice were grouped (*n* = 20 per group) as follows: normal diet + vehicle group (ND), normal diet + calcipotriol group (Cal), 0.1%DDC diet + vehicle group (DDC) and 0.1%DDC diet + low dose calcipotriol group (DDC + Cal). Calcipotriol (80 μg/kg) was administered intraperitoneally daily in calcipotriol-treated groups. Half of the mice in each group were sacrificed after anesthesia by the end of week 2 and the other half were sacrificed by the end of week 4. The livers, kidneys, and serum were collected and stored under −80°C.

### Real-Time Quantitative PCR (RT-qPCR)

Total RNA of mouse tissue or cells was extracted by using TRIzol reagent (Sigma, St. Louis, MO, United States) and then reversed transcribed into cDNA by using the PrimeScript RT Reagent Kit (Takara, Shiga, Japan). The relative gene expression level were measured by real-time PCR on an Applied Biosystems ViiA^TM^ 7 Real-Time PCR System (Applied Biosystems, Foster City, CA, United States) by using SYBR Premix Ex Taq (Takara, Code NO. RR82LR) and expressed by comparative Ct method (△△Ct method). In liver tissue, the expression of Col1A1, α-SMA, vim, TGFβ-1, CK-19, CYP7A1, CYP8B1, MRP2, MRP3, MRP4, OST-α, YAP1, CTGF, ANKRD1 and CCN1 mRNA were measured. In kidney tissue, the expression of MRP2, MRP3, MRP4 and OST-α mRNA were measured. The expression of beta-actin (β-actin) mRNA was used as the endogenous reference control. Primer sequences are listed in Supplementary Table S1.

### Western Blot Analysis

The protein was extracted from liver tissue or cultured cells by using RIPA buffer (Beyotime Institute of Biotechnology, Hangzhou, China). Proteins were separated by using electrophoresis on 6–15% SDS-PAGE gels and transferred to a PVDF membrane. The antibodies used have been listed in subsection “Reagents and Antibodies.” Chemiluminescent substrates (Thermo Scientific) were used for detection.

### Liver Damage Assessment

Serum alanine aminotransferase (ALT), aspartate transaminase (AST), alkline phosphatase (AKP), total bilirubin (TBil), total bile acid (TBA) levels were measured by using the commercially available assay kit purchased from Nanjing Jiancheng Bioengineering Institute (Nanjing, China) according to the manufacturer’s instructions.

### Cell Culture and Treatment

LX2 (a human immortalized HSC line) and RAW264.7, 293T cells were obtained from Cell Bank of Shanghai Institutes of Biological Sciences, Chinese Academy of Sciences. All cell lines were cultured in Dulbecco’s modified Minimal Essential Medium containing 10% fetal bovine serum (FBS), penicillin (100 mg/ml) and streptomycin (100 mg/ml) at 37°C in an atmosphere of 5% CO2. To analyze the role of VDR ligands and MCC950 on NLRP3 expression in LX2 and RAW264.7 cell lines, NLRP3 inflammasome activation was induced with Lipopolysaccharides (LPS; 1 μg/ml) for 3 h followed by adenosine triphosphate (ATP) (5 mM) for 1 h. Then the culture medium was changed and cells were co-cultured with calcipotriol, MCC950 or VDR antagonist calcifediol (Zheng et al., 2018) for 24 h and harvested for total protein and RNA. The doses of calcipotriol and calcipotriol chose for *in vitro* treatment were based on cell counting kit-8 (CCK-8) assay (Supplementary Figures S1A,B). The dose of MCC950 (10 nM) used in *in vitro* treatment was according to previous research (Mridha et al., 2017).

### Plasmids Constructions and shRNAs

Hieff Clone^TM^ One Step Cloning Kit (Yeasen, 10911ES25) was used to clone the ORF sequence of VDR into the pIPFlag-tagged vector with Flag-tag in the N-terminus according to the protocol. The non-targeting control shRNA (shNT) and YAP1-targeting shRNAs used in this research were purchased from Biochemistry and Molecular Cell Biology, Shanghai Jiao Tong University School of Medicine. Primers for plasmids constructions and shRNA sequences were listed in Supplementary Table S2.

### Lentivirus Production and Infection

To product lentivirus, 293T cells were prepared in a 6-cm culture dish with 70%∼80% confluence and then co-transfected with 1.8 μg psPAX2 packaging plasmid, 1.6 μg target plasmid (shRNAs) and 0.6 μg pMD2. G envelope plasmid by using PEI (Polysciences, 24765-1) with serum-free medium for 6–8 h. Six to eight hours later, the serum-free medium was replaced with medium containing 10% FBS and penicillin (100 mg/ml) and streptomycin (100 mg/ml). The transfected 293T cells were incubated at 37°C for 48 h and lentivirus-containing were collected. To construct stable cell lines, target cells were infected with lentivirus supplemented with 5 μg/ml polybrene for 12–24 h and positively selected with puromucin (5 μg/mL) to eliminate uninfected cells for 1 week to generate stable cell lines.

### IP Assays

293T cells were transfected with Flag-VDR for 24 h and then lysed with RIPA lysis buffer for IP (Beyotime Biotechnology, P0013) followed by immunoprecipitation with M2 anti-flag beads (Sigma-Aldrich, A2220). Subsequently, the immunocomplexes were washed with lysis buffer and proteins were analyzed by IB.

### Statistical Analysis

All data were presented as the means ± S.D. Differences between specific groups were determined by using an unpaired Student *t*-test. All statistical analyses were performed using SPSS software package (version 23.0, IBM SPSS) and statistical significance was accepted when *p* < 0.05.

## Results

### Alleviation of Cholestatic Liver Injury by Using Calcipotriol

#### Calcipotriol Alleviate BDL-Induced Cholestasis

**FIGURE 1:**
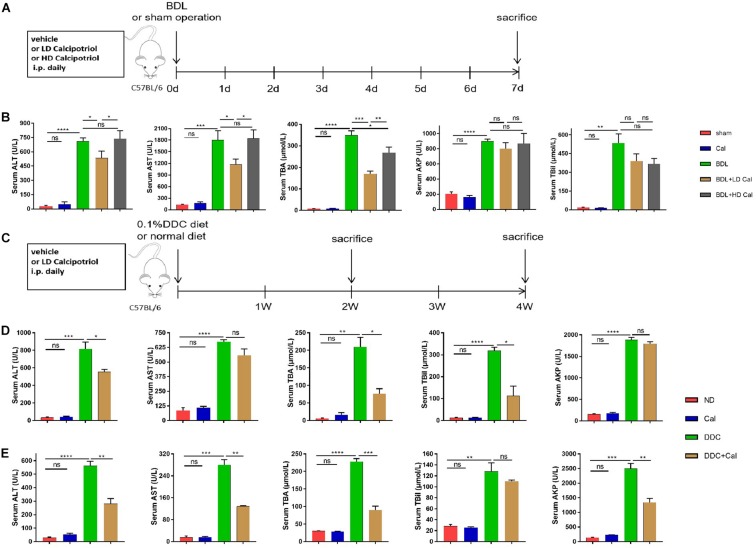
Calcipotriol alleviates bile-duct ligation (BDL)-induced and 3,5-diethoxycarbonyl-1,4-dihydrocollidine (DDC)-induced hepatobiliary injury and cholestasis. **(A,C)** Schematic representation of the experimental design. Two classic methods (BDL and 0.1%DDC diet) were used to establish cholestatic models. Calcipotriol was administered intraperitoneally daily at the dosage [low dose (LD), 80 μg/kg; high dose (HD), 160 μg/kg] shown. **(B)** Serum liver function biomarkers of BDL-induced cholestasis groups. Serum alanine aminotransferase (ALT) and aspartate aminotransferase (AST) levels were significantly lower in BDL + LD Cal mice than those in BDL mice. Serum total BA (TBA) levels decreased notably in both the BDL + LD Cal group and the BDL + HD Cal group compared to those in the BDL group. **(D)** Serum liver function biomarkers of DDC-induced cholestasis groups at 2 weeks. Calcipotriol supplementation led to significant decreases in the serum ALT, total bilirubin (TBil), and TBA levels. **(E)** Serum liver function biomarkers of DDC-induced cholestasis groups for 4 weeks. Serum ALT, AST, AKP, and TBA were significantly lower in DDC + Cal mice compared to those in DDC mice. All data are presented as mean ± SEM (*n* = 5–10, *P < 0.05; **P < 0.01; ***P < 0.001; ****P < 0.0001).

**FIGURE 2:**
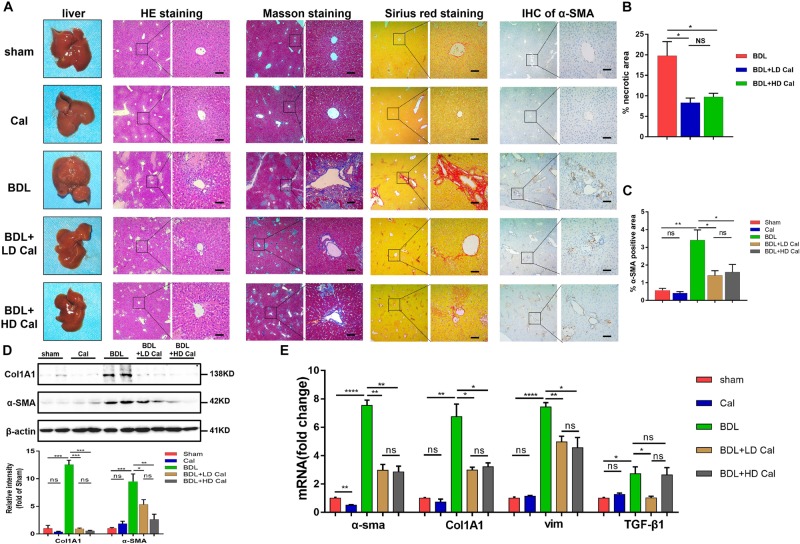
Calcipotriol alleviates BDL-induced liver injury and fibrosis. **(A)** gross picture, hematoxylin and eosin (H&E) staining, Masson staining, Sirius Red staining and Immunohistochemistry (IHC) staining for α-SMA of representative mouse liver samples of Sham, Cal, BDL, BDL + LD Cal, and BDL + HD Cal groups. Scale bar = 100 μm. **(B)** Quantification of necrotic area from H&E sections. **(C)** Image quantification of α-SMA expression. **(D)** Detection of protein expression of α-SMA and Col1A1 in liver samples with Western blot analysis. β-actin was used as the control. Gray scale analysis was performed. **(E)** Detection of mRNA expression of α-SMA, Col1A1, vim, and TGF-β1 in liver samples with real-time polymerase chain reaction (RT-PCR) normalized against β-actin and expressed as 2-ΔΔCT. All data are presented as mean ± SEM (*P < 0.05; **P < 0.01; ***P < 0.001; ****P < 0.0001).

To explore the protective role of calcipotriol in BDL-induced cholestasis, calcipotriol was administered intraperitoneally daily. The experimental procedure and drug dosages used are concisely illustrated in Figure 1A. The liver function and integrity are assessed in term of liver function biomarkers (serum ALT, AST, AKP, TBile, and TBA). As expected, all liver function biomarkers increase notably in the BDL group (Figure 1B). Calcipotriol administration accompanied with a Sham-operated had no influenced on the levels of serum markers (Figure 1B). LD calcipotriol supplement decreased significantly the serum ALT and AST in BDL-induced cholestasis, but HD calcipotriol administration did not (Figure 1B). LD is superior to HD in reducing serum biomarkers of liver injury. This appeared to be due to the optimal dosage and side effect of calcipotriol in the treatment of BDL-induced cholestasis in mice which needs further study. Moreover, both LD and HD calcipotriol supplement reduced the level of serum TBA (Figure 1B). Meantime, there was no significant difference in AKP and TBil between calcipotriol administration groups and BDL group (Figure 1B). Notably, the liver gross pictures and H&E staining of the liver sections confirmed the biochemical results. The area and number of visible necrotic lesions on the surface of the liver are significantly reduced by both LD and HD calcipotriol supplement (Figure 2A). HE staining of the liver tissues confirmed the protective role of calcipotriol in BDL-induced cholestasis evidenced by decreased area of focal coagulative necrosis and inflammation infiltration (Figure 2B).

#### Calcipotriol Alleviate DDC-Induced Cholestasis

**FIGURE 3:**
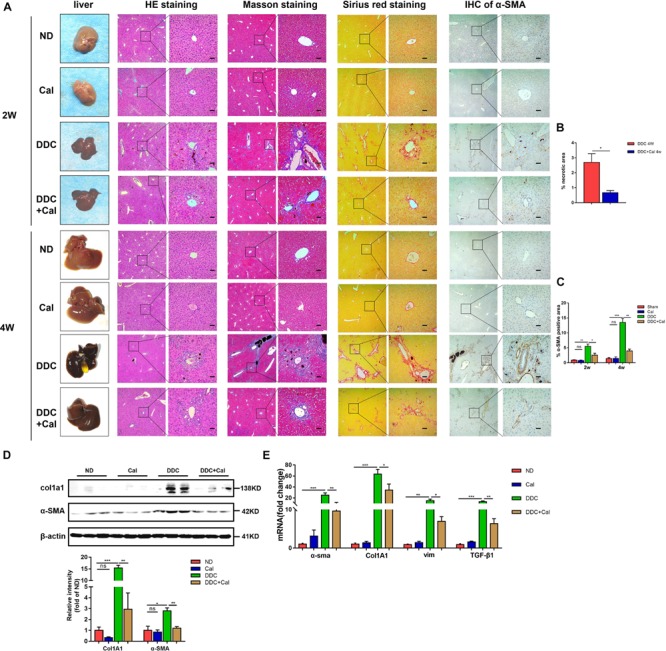
Calcipotriol alleviates DDC-induced liver injury and fibrosis. **(A)** Gross picture, hematoxylin and eosin (H&E) staining, Masson staining, Sirius Red staining and Immunohistochemistry (IHC) staining for α-SMA of representative mouse liver samples of ND, Cal, DDC, DDC + Cal at 2 and 4 weeks. Scale bar = 100 μm. **(B)** Quantification of necrotic area from H&E sections. **(C)** Image quantification of α-SMA expression. **(D)** Detection of protein expression of α-SMA and Col1A1 in liver samples with Western blot analysis. β-Actin was used as the control. Gray scale analysis was performed. **(E)** Detection of mRNA expression of α-SMA, Col1A1, vim, and TGF-β1 in liver samples with RT-PCR normalized against β-actin and expressed as 2-ΔΔCT. All data are presented as mean ± SEM (^∗^P < 0.05; ^∗∗^P < 0.01; ^∗∗∗^P < 0.001).

To characterize the role of calcipotriol in DDC-induced cholestatic liver injury, calcipotriol was administered intraperitoneally daily for 2 and 4 weeks. The experimental procedure was briefly illustrated in Figure 1C. All hepatic serum biomarkers of mice fed with 0.1% DDC diet for 2 and 4 weeks increased notably compared with those of ND group suggesting that clear-cut cholestasis was induced in this model (Figures 1D,E). Calcipotriol supplement reduced the levels of serum ALT, TBA and TBil of DDC-induced cholestasic mice for 2 weeks and decreased the levels of serum ALT, AST, TBA and AKP for 4 weeks (Figures 1D,E). Meantime, according to HE staining (Figure 3A), there was marked architectural distortion of the lobular structure and a notable increase in area of necrosis evidenced by quantification (Figure 3B) in DDC-induced induced choletasis. Calcipotriol treatment reduced these pathological changes compared with the DDC group (Figures 3A,B).

### Prevention of Cholestasis-Induced Liver Fibrosis Development in Mice by Calcipotriol

**FIGURE 4:**
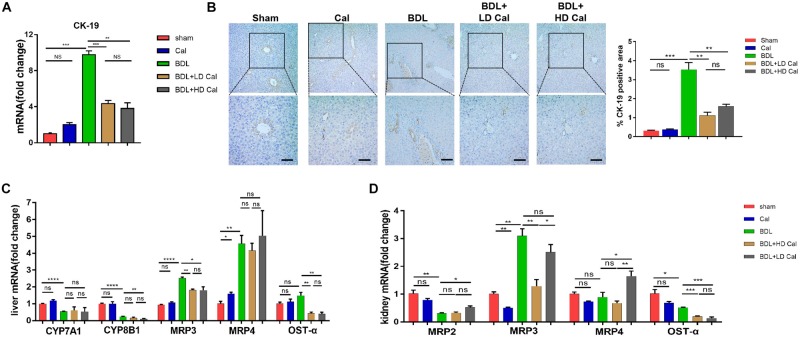
Calcipotriol inhibits ductular reaction and modulates synthesis and transport of BA. **(A)** Detection of mRNA expression of CK-19 in liver samples with RT-PCR. **(B)** IHC staining of representative mouse liver samples for CK-19 and image quantification of CK-19 expression. Scale bar: 100 μm. **(C)** Detection of mRNA expression of CYP7A1, CYP8B1, MRP3, MRP4 and OST-α in liver samples with RT-PCR. **(D)** Detection of mRNA expression of MRP2, MRP3, MRP4, and OST-α in kidney samples with RT-PCR. All RT-PCR results were normalized against β-actin and expressed as 2-ΔΔCT. All data are presented as mean ± SEM (*P < 0.05; **P < 0.01; ***P < 0.001; ****P < 0.0001).

The effect of calcipotriol in cholestasis-induced fibrogenesis was explored by performing Masson staining and Sirius Red staining (specific staining for ECM) of liver sections. As expected, Masson staining and Sirius Red staining demonstrated notable onion-like fibrotic lesions and porto-portal bridging fibrosis in both the BDL group and the DDC group (Figures 2A, 3A). A comparison of the two model groups revealed that calcipotriol treatment ameliorated cholestasis-induced fibrosis, as evidenced by reduced collagen deposition (Figures 2A, 3A). In addition, immunohistochemistry (IHC) staining of alpha-smooth muscle actin (α-SMA), a unique marker of HSC activation, indicated strong periductal expression in cholestasis, which was downregulated in the calcipotriol-treated groups (Figures 2A,C, 3A,C). Moreover, IHC staining of Col1A1, another pro-fibrotic marker, demonstrated an increase in Col1A1 in periductal area in both DDC- and BDL-induced choletasis. Calcipotriol treatment reversed the increase of Col1A1 expression (Supplementary Figures S2A,B). Meantime, Western blot was performed to determine the protein levels of the pro-fibrotic markers (α-SMA and Col1A1). The liver tissue protein levels of these two pro-fibrotic makers decreased significantly upon calcipotriol supplementation in cholestasis compared to those of the two model groups (Figures 2D, 3D), which further confirmed the observations made post histological staining. RT-PCR demonstrated an notable decrease in mRNA levels of pro-fibrotic markers (α-SMA, Col1A1, vim and TGF-β1) by calcipotriol treatment in both BDL- and DDC-induced cholestasis (Figures 2E, 3E). Moreover, IHC staining and RT-PCR of cytokeratin-19 (CK-19) were performed to analyze its expression level. CK-19 is an antigenic marker of biliary epithelial cells and bile ductules. CK-19 overexpression is a hallmark of cholangiocyte proliferation and ductular reaction (DR) (Junge et al., 2017). DR involving both hepatic progenitor cells and the extracellular matrix is observed in response to acute severe and chronic liver injury, and it acts as a liver-repair mechanism. However, excessive DR contributes to liver structure derangement and fibrogenesis (Desmet, 2011). As illustrated in Figures 4A,B, BDL significantly elevated CK-19 levels, and both LD and HD calcipotriol supplementation reversed this elevation.

### Reduction of Serum Bile Acid by Calcipotriol Through Regulation of Bile Acid Transporters and Synthetases

Cholestasis causes changes in the expression of the genes involved in BA metabolism and transport. Because calcipotriol significantly reduced serum BA levels in cholestasis, we tested the effect of calcipotriol on BA metabolism and transport. CYP7A1, which serves as the rate-limiting enzyme in the classical BA synthesis pathway, and CYP8B1 were markedly downregulated in BDL-induced cholestasis. Calcipotriol supplementation significantly reduced CYP8B11 mRNA expression in BDL + HD Cal mice compared to that in BDL mice, but it had no effect on CYP7A1 (Figure 4C). Although a previous study showed that vitamin D3 can reduce CYP7A1 expression (Ogura et al., 2009), this inconsistent experimental results is probably due to the different experimental conditions and types of VDR ligands. BDL notably raised the mRNA level of MRP3, a basolateral efflux pump, thus contributing to BA and bilirubin efflux into blood circulation. This result is consistent with the concept that the basolateral efflux pumps of hepatocytes compensate for the impaired canalicular efflux of compounds into bile in cholestasis. Intriguingly, calcipotriol significantly suppressed the mRNA level of MRP3 (Figure 4C), which is contrary to the regulation of vitamin D3 on MRP3, as reported in a previous study (Ogura et al., 2009). Moreover, calcipotriol elevated MRP4 mRNA expression, but had no effect on the regulation of MRP4 in BDL- induced cholestasis (Figure 4C). Meantime, a significant reduction in the mRNA level of OST-α, a basolateral bidirectional transporter, in BDL + Cal group compared with BDL group (Figure 4C). Moreover, the protein levels of CYP7A1, CYP8B1 and MRP3 in liver were detected by conducting western blot which confirmed the results of RT-PCR (Supplementary Figure S3). We also tested the mRNA levels of BA transporters in the kidney. The BA transporters MRP2, MRP3, MRP4, and OSTα are distributed in renal tubular cells and are involved in BA transport. In cholestasis, urine excretion of BA through these transporters is the major route for eliminating excess toxic BA. As shown (Figure 4D), BDL significantly raised the renal expression of MRP3 but downregulated the renal expression of MRP2 and OSTα. In BDI-induced cholestasis, HD calcipotriol supplementation remarkably increased the mRNA expression of MRP2 and MRP4, two BA transporters that are expressed apically and localized primarily in proximal tubules. By contrast, calcipotriol supplementation reduced the expression of the basolateral BA transporters MRP3 and OST-α (Figure 4D).

### Alleviation of Cholestatic Liver Injury and Fibrosis by Calcipotriol Through NLRP3 Inhibition

**FIGURE 5:**
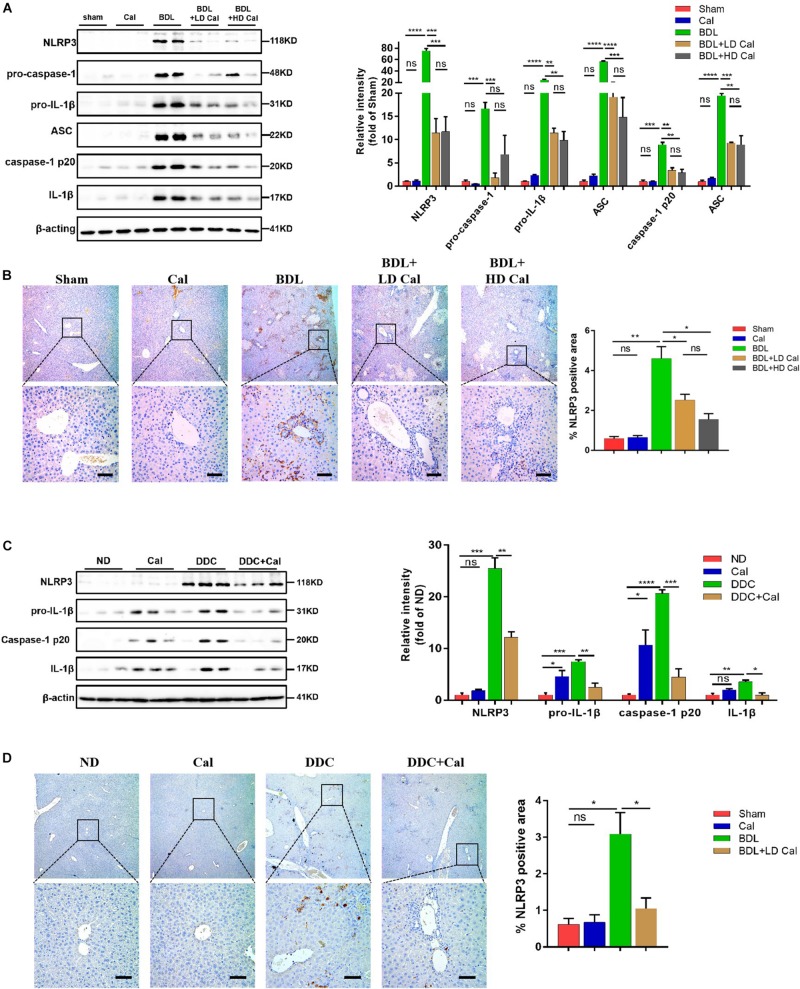
Calcipotriol supplement inhibits the NLRP3 inflammasome activation during DDC-induced and BDL-induced cholestatic liver injury. **(A)** Detection of protein levels of NLRP3, pro-caspase 1, pro-IL-1β,ASC, caspase-1 p20, and IL-1β in representative mouse liver samples of Sham, Cal, BDL, BDL + LD Cal, and BDL + HD Cal groups with Western blot analysis. β-Actin was used as the control. Gray scale analysis was performed to determine relative proportions of NLRP3, pro-caspase 1, pro-IL-1β,ASC, caspase-1 p20, and IL-1β. **(B)** IHC staining of representative mouse liver samples of Sham, Cal, BDL, BDL + LD Cal, and BDL + HD Cal groups for NLRP3. Scale bar = 100 μm. Image quantification of NLRP3 expression was performed. **(C)** Detection of protein levels of NLRP3, pro-IL-1β, caspase-1 p20, and IL-1β in representative mouse liver samples of ND, Cal, DDC and DDC + Ca groups with Western blot analysis. β-Actin was used as the control. Gray scale analysis was performed to determine relative proportions of NLRP3, pro-IL-1β, caspase-1 p20, and IL-1β. **(D)** IHC staining of representative mouse liver samples of ND, Cal, DDC and DDC + Ca groups for NLRP3. Scale bar = 100 μm. Image quantification of NLRP3 expression was performed. All Bars represent mean ± SEM (*P < 0.05; **P < 0.01; ***P < 0.001; ****P < 0.0001).

**FIGURE 6:**
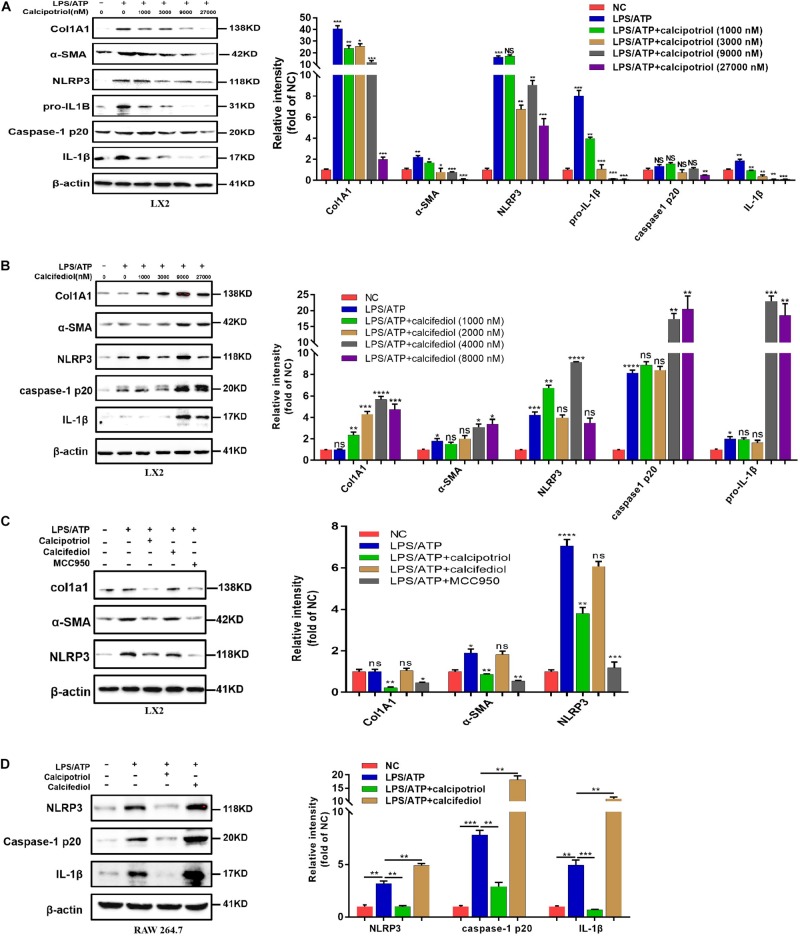
Calcipotriol retards liver fibrogenesis by inhibiting the HSC activation through inhibiting NLRP3 activation. **(A)** Stimulation of LPS-primed LX2 cells with ATP for 1 h. Thereafter, the cells were treated with different concentrations of VDR agonist calcipotriol for 24 h. The protein levels of Col1A1, α-SMA, NLRP3, pro-IL-1β, caspase-1 p20, and IL-1β were detected by conducting Western blot analysis. β-Actin was used as the control. **(B)** Stimulation of LPS-primed LX2 cells with ATP for 1 h. Thereafter, the cells were treated with different concentrations of VDR antagonist calcifediol for 24 h. The protein levels of Col1A1, α-SMA, NLRP3, caspase-1 p20, and IL-1β were detected by conducting Western blot analysis. β-Actin was used as the control. **(C)** Stimulation of LPS-primed LX2 cells with 1 mM ATP for 1 h. Thereafter, the cells were treated with vehicle, VDR agonist calcipotriol, VDR antagonist calcifediol, and NLRP3 inhibitor MCC950 separately for 24 h. Western blot analysis of Col1A1, α-SMA, NLRP3 in cells. β-actin was used as the control. **(D)** Stimulation of LPS-primed RAW264.7 cells with 1 mM ATP for 1 h. Thereafter, the cells were treated with vehicle, VDR agonist calcipotriol, VDR antagonist calcifediol separately for 24 h. Western blot analysis of NLRP3, caspase-1 p20, and IL-1β in cells. β-actin was used as the control. Gray scale analysis of Western blot was performed and all bars represent mean ± SEM (*P < 0.05; **P < 0.01; ***P < 0.001; ****P < 0.0001).

A previous study demonstrated that the NLRP3 pathway plays an important role in the development of liver injury and damage in cholestasis (Yang et al., 2018). Our recent study confirmed that the inhibition of NLRP3 by using its selective inhibitor MCC950 alleviated cholestatic liver injury and fibrosis in mice. Inzaugarat et al. (2019) pointed out that NLRP3 inflammasome activation aggravated liver fibrosis, and the NLRP3 signal served as an independent pathway in HSC activation and liver fibrosis development. Many clinical and fundamental studies on the anti-inflammatory and anti-fibrotic effects of VDR strongly associated VDR with the NLRP3 pathway, which is essentially an inflammation-related pathway. In our *in vivo* study, the protein levels of the NLRP3 inflammasome and the downstream effectors of the canonical NLRP3 pathway (ASC, pro-caspase1, capase1, pro-IL-1β, and IL-1β) increased significantly in the BDL group and DDC group compared to those in the Sham group and ND group, and calcipotriol supplementation reversed this increase (Figures 5A,C). The result was verified by liver IHC staining for NLRP3 (Figures 5B,D). To obtain direct evidence that VDR ligands can affect liver fibrosis and injury by controlling the NLRP3 pathway, the LX2 cell line (a human-immortalized HSC line) and the RAW264.7 cell line (a mouse-immortalized macrophage line) were used to determine the role of VDR ligands in modulating the NLRP3 signal. As expected, the activation of NLRP3 through LPS/ATP stimulation promoted HSC activation, as indicated by the overexpression of α-SMA and Col1A1 (Figure 6A). Supplementation of VDR agonist calcipotriol inhibited the protein level of NLRP3 and its downstream effectors in a dose-dependent manner, accompanied by lowering of pro-fibrotic markers (Figure 6A). By contrast, the VDR antagonist calcifediol had the opposite effect (Figure 6B). To demonstrate that NLRP3 inhibition can inhibit HSC activation, MCC950, a selective NLRP3 inhibitor, was administered after LPS/ATP stimulation. The inhibition of NLRP3 by using MCC950 significantly inhibited HSC activation, as evidenced by the decreases in pro-fibrotic markers (Figure 6C). In the liver, Kupffer cell, a type of hepatic macrophage, and recruited liver macrophages together contribute to the augmentation and aggravation of hepatic inflammation to promote liver injury. Moreover, activation of the NLRP3 inflammasome in macrophages induces excessive extracellular secretion of matured IL-1β, the main end product of the NLRP3 signal, which promotes liver fibrosis and HSC activation by binding to the IL-1β receptor on the HSC membrane (Pradere et al., 2013; Czaja and Amir, 2017). Stimulated by LPS/ATP, the protein levels of NLRP3 and downstream effectors increased, and supplementation with VDR ligands modulated the NLRP3 pathway (Figure 6D), which is consistent with the results obtained using the HSC line. Put together, these data suggest that VDR activation by calcipotriol alleviates liver fibrosis and injury in cholestasis through inhibition of NLRP3 signal, and calcipotriol inhibits HSC activation by inhibiting NLRP3 pathway both directly and indirectly.

### Inhibition of NLRP3 Inflammasome by Calcipotriol Through Activation of YAP1

**FIGURE 7:**
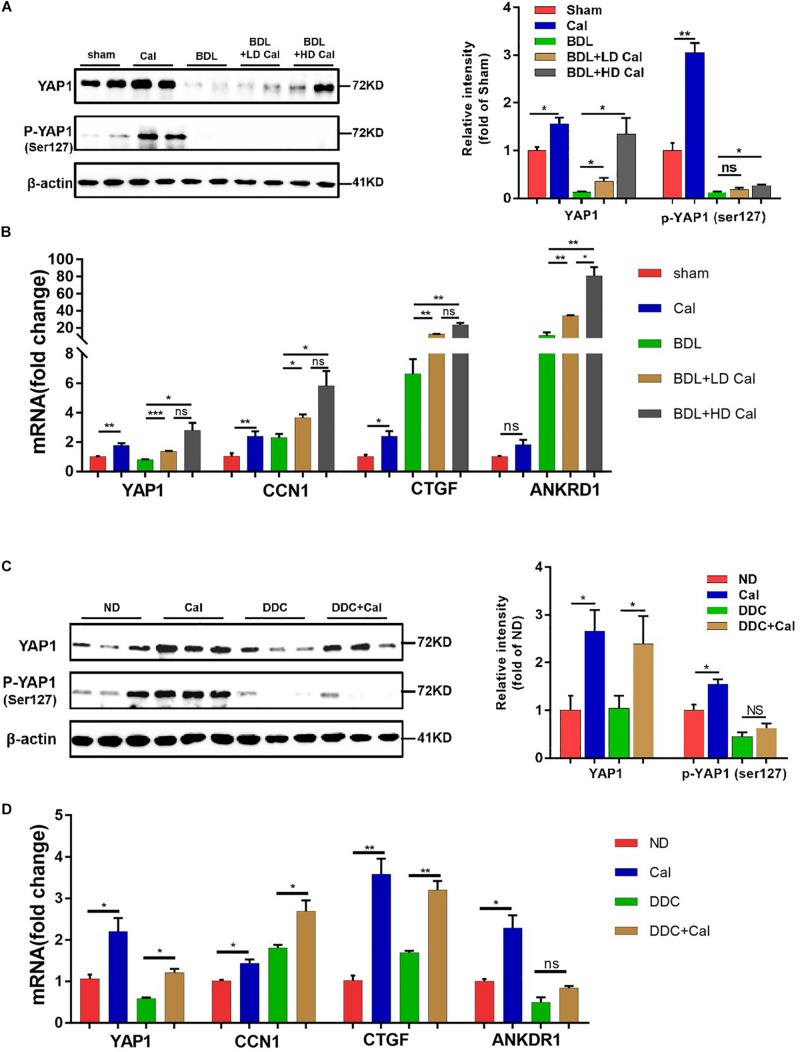
Calcipotriol supplement enhance yes-associated protein 1 (YAP1) expression during cholestatic liver injury. **(A)** Increase in the levels of both YAP1 and p-YAP1 (ser127) in representative mouse liver samples of Sham, Cal, BDL, BDL + LD Cal, and BDL + HD Cal groups upon calcipotriol supplementation. Protein levels were detected by conducting Western blot analysis and β-actin was used as the control. Gray scale analysis was performed to determine relative proportions of YAP1 and p-YAP1 (ser127). **(B)** Elevation of the mRNA levels of YAP1 and its target genes (CTGF, ANKRD1, and CCN1) upon calcipotriol supplementation in representative mouse liver samples of Sham, Cal, BDL, BDL + LD Cal, and BDL + HD Cal groups. mRNA expression was detected with RT-PCR. The results were normalized against β-actin and expressed as 2-ΔΔCT. **(C)** Increase in the levels of both YAP1 and p-YAP1 (ser127) in representative mouse liver samples of ND, Cal, DDC and DDC + Cal groups upon calcipotriol supplementation evidenced by conducting Western blot analysis and β-actin was used as the control. Gray scale analysis was performed to determine relative proportions of YAP1 and p-YAP1 (ser127). **(D)** Elevation of the mRNA levels of YAP1 and its target genes (CTGF, ANKRD1, and CCN1) upon calcipotriol supplementation in representative mouse liver samples of ND, Cal, DDC and DDC + Cal groups. mRNA expression was detected with RT-PCR. The results were normalized against β-actin and expressed as 2-ΔΔCT. All data are presented as mean ± SEM (*P < 0.05; **P < 0.01; ***P < 0.001).

**FIGURE 8:**
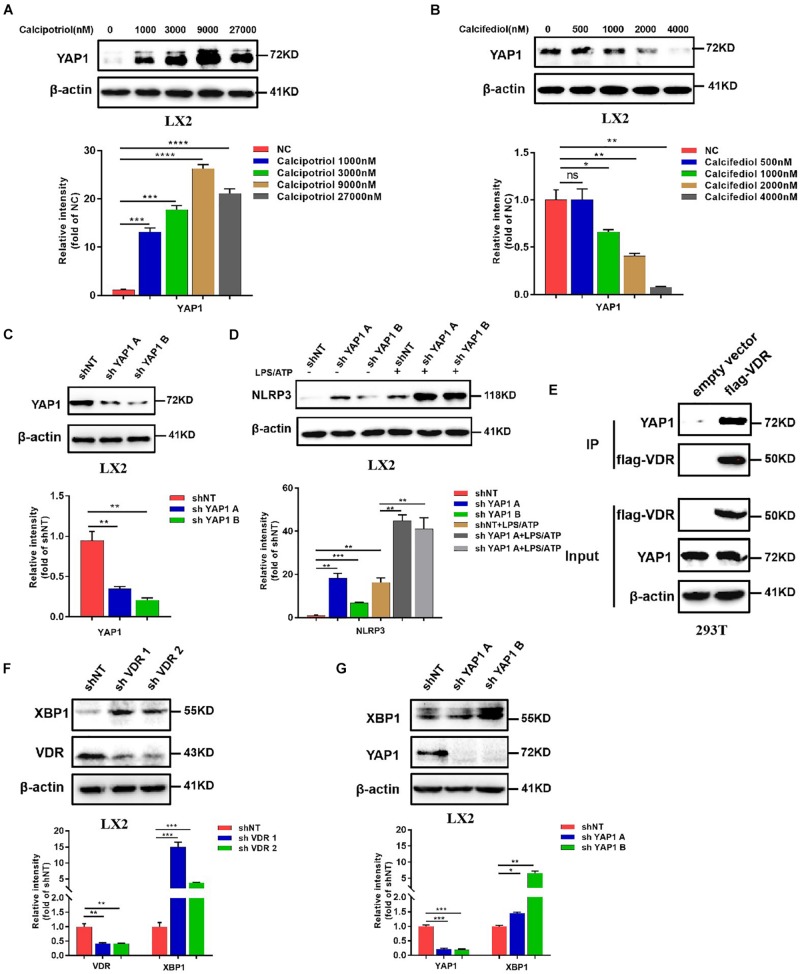
Calcipotriol inhibits NLRP3 by activating yes-associated protein 1 (YAP1). **(A)** Treatment of LX2 cells with different concentrations of calcipotriol for 24 h. YAP1 in the cells was detected by conducting Western blot analysis, and β-actin was used as the control. VDR agonist calcipotriol supplementation increased the YAP1 level. **(B)** Treatment of LX2 cells with different concentrations of calcifediol for 24 h. YAP1 in the cells was detected by conducting Western blot analysis, and β-actin was used as the control. VDR antagonist calcifediol supplementation increased the YAP1 level. **(C)** Knockout of YAP1 by shRNA in LX2 cells. Knockout efficiency was verified based on the results of the Western blot analysis. β-actin was used as the control. **(D)** In LX2 cells, YAP1 knockout by shRNA increased NLRP3 activation and promoted sensitivity to LPS/ATP-induced NLRP3 activation. The protein level of NLRP3 was detected by conducting Western blot analysis. **(E)** Co-IP of VDR and YAP1 for assessing their protein–protein interaction in 293T cells. **(F)** In LX2 cells, VDR knockout by shRNA increased XBP1 expression. The protein level of NLRP3 was detected by conducting Western blot analysis, and knockout efficiency was verified based on the results of the Western blot analysis. β-actin was used as the control. **(G)** In LX2 cells, YAP1 knockout by shRNA increased XBP1 expression. The protein level of NLRP3 was detected by conducting Western blot analysis, and knockout efficiency was verified based on the results of the Western blot analysis. β-actin was used as the control. Gray scale analysis of Western blot was performed and all data were present as mean ± SEM (*P < 0.05; **P < 0.01; ***P < 0.001).

Our *in vivo* study revealed that the protein levels of YAP1 in liver tissue decreased markedly in cholestasis (Figures 7A,C), which may possibly be ascribed to the effect of FXR, the major nuclear receptor involved in BA metabolism (Anakk et al., 2013). By contrast, calcipotriol supplementation significantly raised the level of YAP1 under both normal and cholestatic conditions (Figures 7A,C). Meantime, the protein level of VDR detected by western blot was downregulated in DDC-induced cholestasis and calcipotriol supplement did not revise this downregulation (Supplementary Figure S3). Because YAP1 phosphorylation increases its cytoplasmic retention and inhibits its function as a transcriptional co-activator, we also detected changes in the phosphorylation levels of YAP1 (Ser127), which were consistent with the changes in YAP1 protein levels under both normal and cholestatic conditions (Figures 7A,C). Moreover, RT-PCR was performed to analyze the mRNA levels of YAP1 and the downstream target genes of YAP1, including the connective tissue growth factor (CTGF), ankyrin repeat domain 1 (ANKRD1), and cellular communication network factor 1 (CCN1). The results of RT-PCR demonstrated that VDR activated by calcipotriol increased YAP1 expression by regulating the transcriptional level of YAP1 rather than altering its phosphorylation modification (Figures 7B,D). Furthermore, we treated LX2 cell line with different concentrations of calcipotriol for 24 h. The protein levels of YAP1 increased notably in the calcipotriol-treated cells compared to that in the vehicle-treated cells (Figure 8A). By contrast, treatment with the VDR antagonist calcifediol significantly reduced YAP1 expression dose-dependently (Figure 8B). To further explore the role of YAP1 in modulating NLRP3 activation, a stable low expression of the YAP1 LX2 cell line was obtained by infecting cells with lentivirus. The YAP1 expression level was detected by conducting western blot analysis (Figure 8C). YAP1 knockout increased NLRP3 expression remarkably and sensitized LX2 cells to LPS/ATP-induced NLRP3 activation (Figure 8D). It is well acknowledged that both VDR and YAP1 are located in the cytoplasm and translocated into the nucleus to function as transcription factors after activation. Co-immunoprecipitation (Co-IP) were performed to explore the interaction of YAP1 and VDR in 293T cell line. The results demonstrated that VDR could interact with YAP1 (Figure 8E). The study of Li et al. (2019) demonstrated that YAP1, a key downstream effector of the Hippo pathway, could interact with β-catenin and colocalize in the nucleus to downregulate the target gene X-box binding protein 1 (XBP1) leading to reduced NLRP3/caspase-1 activity. To explore whether VDR could interact with YAP1 to regulate XBP1, VDR^–/–^ LX2 cells and YAP1^–/–^ LX2 cells were obtained by infecting cells with lentivirus. It is highly surprising that both YAP1 and VDR knockdown led to a notable expression of XBP1 (Figures 8F,G), indicating that XBP1 may severs as a downstream effector of VDR and YAP1 to regulate NLRP3 pathway which still needs in-depth researched. In conclusion, we proved that calcipotriol inhibited NLRP3 expression by activating YAP1.

**FIGURE 9:**
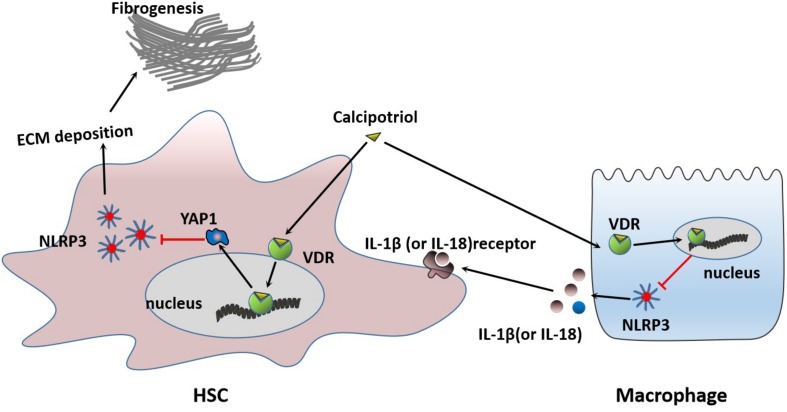
Proposed mechanisms of calcipotriol-induced inhibition of the NLRP3 pathway through activation of YAP1 to alleviate liver injury and fibrosis.

## Discussion

In our study, we demonstrated that calcipotriol could alleviate cholestatic liver injury and fibrosis by inhibiting the NLRP3 pathway. Calcipotriol supplementation suppressed HSC activation induced by NLRP3 activation. Moreover, we proved that VDR is closely associated with the NLRP3 signal. VDR ligands influence the regulation of NLRP3 by regulating YAP1 expression. VDR activation could induce YAP1 expression to influence the inhibition of NLRP3 inflammasome activation.

Cholestasis is a common clinical syndrome caused by the defective secretion of bile owing to diverse etiologies, such as extrahepatic biliary obstruction due to biliary stones, tumor, or atresia and intrahepatic cholestasis due to viruses, alcohol, drugs, or autoimmune diseases. Without effective treatment, severe cholestasis may lead to hepatocellular impairment, liver fibrosis, or eventually cirrhosis, hepatic failure, and carcinoma. A growing body of evidence indicates that the NLRP3 inflammasome is significantly activated and plays an important role in cholestatic liver injury and fibrosis (Gong et al., 2016; Hao et al., 2017; Qu et al., 2019). Liver damage and fibrosis due to cholestasis can be alleviated by using NLRP3 inhibitors or by NLRP3 knockout *in vivo* (Ma et al., 2018; Yang et al., 2018; Qu et al., 2019).

VDR, a member of the nuclear receptor superfamily, is a crucial regulator of mineral and bone homeostasis. In the liver, VDR expression in non-parenchymal cells (HSC, macrophages, and biliary epithelial cells) is considerably stronger than that in parenchymal cells (Gonzalez-Sanchez et al., 2017). Multiple previous literatures have confirmed that the activation state of VDR can modulate the functions of non-parenchymal and parenchymal liver cells, such as fibrogenesis, integrity of biliary epithelial cells, liver cancer, and BA metabolism. Recently, two studies published in Hepatology further emphasized the importance of VDR in regulating inflammatory response, and VDR activation by VDR ligands may be useful as a potential method for treating multiple liver diseases (Dong et al., 2019; Zhou et al., 2019). In previous studies, vitamin D3 has been proved to abate fibrosis and modulate BA regulatory genes in BDL-induced cholestasis (Ogura et al., 2009). Due to the effect of vitamin D3 on calcium metabolism, the interest in clinical application remained poor for years. However, calcipotriol, a potent VDR agonist and vitamin D3 analog, has no effect on calcium metabolism which may make it more clinically useful. In our study, calcipotriol was first applied to two classic cholestatic mouse models to test its therapeutic efficiency. Moreover, we demonstrated in a complementary manner the anti-inflammation and anti-fibrosis effect of VDR through NLRP3 inhibition for the first time.

The Hippo pathway is a highly conserved protein kinase cascade that has been proved to regulate cell fate, cell growth, tissue growth, tumorigenesis, and tumor metastasis (Guo et al., 2016; Yui et al., 2017; Oh et al., 2018). As the key downstream effector of hippo pathway, YAP1 has been demonstrated to have a close linkage with members of the nuclear receptor superfamily, such as FXR, PXR, and RXR (Anakk et al., 2013; Deng et al., 2019; Jiang et al., 2019). During cholestatic liver injury, multiple factors, such as BA (Gong et al., 2016) and endotoxemia (Hao et al., 2017), contribute to the activation of NLRP3 which represents a host defense to pathogens and damaging signals. However, excessive NLRP3 inflammasome activation, which contributes to inflammatory liver injury and development of liver fibrosis should be tightly controlled (Jin et al., 2019). Our research combined with previous studies proved that VDR and YAP1 was extremely downregulated in cholestasis (Anakk et al., 2013; Gonzalez-Sanchez et al., 2017) and both VDR^–/–^ mice and YAP1^–/–^ mice were more sensitive to cholestasis-induced liver injury (Bai et al., 2012; Firrincieli et al., 2013). Two recent studies proved that YAP1 activation could attenuate hepatic damage and fibrosis in liver ischemia-reperfusion injury and inhibit NLRP3 activation (Li et al., 2019; Liu et al., 2019). In our research, VDR agonist Calcipotriol could negatively regulate NLRP3 inflammation activation both *in vivo* and *in vitro*. Calcipotriol supplement revised the YAP1 downregulation in cholestasis but did not effect the expression of VDR. Calcipotriol may work to counteract metabolic inflammation via activating VDR and overexpession of YAP1 to suppress the excessive activation of NLRP3 inflammasome in cholestasis. Meantime, our studies found that VDR was able to interact with YAP1 and both VDR knockout and YAP1 knockout led to an overexpression of XBP1, which was recognized to lead to increased NLRP3/caspase-1 activity by previous research (Yue et al., 2016; Li et al., 2019). Whether XBP1 acts as a downstream effector of VDR and YAP1 to regulate NLRP3 pathway still needs further study.

Liver fibrosis results from sustained scarring responses to acute or chronic liver injuries caused by various pathological factors which can disrupt the normal structure of the liver and liver function, leading eventually to liver cirrhosis (Yang et al., 2017). HSC is the main fibrogenic cell involved in the progress of liver fibrosis. HSC activation-transdifferentiation of quiescent cells into fibrogenic myofibroblasts, together with excess ECM production, plays an important role in the initiation and development of liver fibrosis. Even as fibrosis-related signals and mediators continue to emerge, the mechanism of HSC activation remains controversial (Tsuchida and Friedman, 2017). Recently, a study highlighted the direct role of the NLRP3 inflammasome in HSC activation, which directly triggers liver fibrosis (Inzaugarat et al., 2019). The vital role of VDR as an anti-inflammatory and anti-fibrotic strongly associates VDR with the NLRP3 pathway, a critical pathway mediating inflammatory response. In this study, we unveiled a new mechanism by which VDR protects the liver from injury and fibrosis and inhibits HSC activation by inhibiting the NLRP3 signal. Meantime we found that calcipotriol supplement could work to inhibit NLRP3 inflammasome activation in cholestasis through counteracting the extreme downregulation of YAP1 to alleviate live injury and fibrosis. Multiple researches recognized YAP1 as an pro-fibrogenic factor, however due to different liver disease models, the role of YAP1 in hepatic anti-fibrosis may be controversial. YAP1 activation was previously recognized as an important role to promote liver regeneration and alleviate liver injury in cholestasis by two studies (Bai et al., 2012; Tharehalli et al., 2018). Besides a resent study has shown that activation of YAP could attenuate hepatic damage and fibrosis in liver ischemia-reperfusion injury (Liu et al., 2019). It is highly likely that YAP activation plays an important role in hepatic protection, regeneration, anti-fibrosis in acute liver injury. Notably, we found that VDR activation can induce YAP1 expression at the transcriptional level in liver tissue and HSC line and increased YAP1 further exerts its inhibitory effect on NLRP3 expression to alleviate cholestatic liver fibrosis and injury. We reported for the first time that VDR ligands can modulate YAP1 expression at the transcriptional level by changing the activation state of VDR. YAP1 proved to be an important co-regulator of tissue growth, tissue development, tumorigenesis, and tumor metastasis. Further studies are urgently needed to determine whether VDR ligands have any effects in these areas.

## Conclusion

We demonstrated that calcipotriol alleviates cholestatic liver injury and fibrosis through the inhibition of NLRP3 inflammasome activation by activating YAP1. The results of our study highlight the potential of calcipotriol as a promising therapeutic strategy for cholestasis. Furthermore, fundamental research and clinical trials of existing VDR ligands used to treat cholestasis are necessary, and further elucidation of the mechanisms of VDR-involved signals may be crucial for treating liver-related disorders.

## Data Availability Statement

The datasets generated for this study are available on request to the corresponding author.

## Ethics Statement

The animal study was reviewed and approved by the Ethics Committee of Renji Hospital.

## Author Contributions

XW, JQ, and KL: conceptualization. XW: methodology. XW, GW, and JQ: validation. XW: formal analysis. GW: resources. GW and JQ: data curation. XW: writing—original draft preparation. XW, ZY, and RP: writing—review and editing. GW and JQ: visualization. KL: supervision and project administration. GW: funding acquisition.

## Conflict of Interest

The authors declare that the research was conducted in the absence of any commercial or financial relationships that could be construed as a potential conflict of interest.
