# Supplementary material for: Calcipotriol Inhibits NLRP3 Signal Through YAP1 Activation to Alleviate Cholestatic Liver Injury and Fibrosis
Source: Front Pharmacol. 2020 Mar 31;11:200. doi: 10.3389/fphar.2020.00200 (PMC7136474; doi:10.3389/fphar.2020.00200)
Supplement: Supplementary file 1 [file Data_Sheet_1.docx]

**Supplemental Table 1.** **Primers for Quantitive RT-PCR Analysis.**

| gene | Full name | ID | Sequences(Forward/Reverse 5’-3’) |
| --- | --- | --- | --- |
| β-actin | beta-actin | 11461 | AGCCATGTACGTAGCCATCC  CTCTCAGCTGTGGTGGTGAA |
| α-SMA | alpha-smooth muscle actin | 11475 | CTGACAGAGGCACCACTGAA  CATCTCCAGAGTCCAGCACA |
| Col1A1 | Collagen type I alpha 1 | 12842 | GAGCGGAGAGTACTGGATCG  GCTTCTTTTCCTTGGGGTTC |
| vim | vimentin | 22352 | CAGATGCGTGAGATGGAAGA  TCCAGCAGCTTCCTGTAGGT |
| TGF-β1 | transforming growth factor beta 1 | 21803 | TTGCTTCAGCTCCACAGAGA  TGGTTGTAGAGGGCAAGGAC |
| CYP7A1 | cytochrome P450, family 7, subfamily a, polypeptide 1 | 13122 | ACACCATTCCTGCAACCTTC  GCTGTCCGGATATTCAAGGA |
| CYP8B1 | cytochrome P450, family 8, subfamily b, polypeptide 1 | 13124 | AACTGATGGCTCACCCACACTT  ATCGAGTAAGGCAGGCTGGAT |
| MRP2 | multi-drug related protein-2 | 12780 | GCTTCCCATGGTGATCTCTT  CTTGGATTGTGGCTTCCAAG |
| MRP3 | multi-drug related protein-3 | 76408 | CCCTGCTCCTGTCTTCTTTG  GCTGAGAGGATCTTGGAACG |
| MRP4 | multi-drug related protein-4 | 239273 | CATACCATTGGTTCCGCTCT  TGCATCAAACAGCTCCTGAC |
| OST-α | organic solute transporter α | 106407 | GGCATCTATGACCCAGGAGA  GGTCAGGATGACAAGCACCT |
| YAP1 | yes-associated protein 1 | 22601 | AAGGAGAGACTGCGGTTGAA  TGCTCCAGTGTAGGCAACTG |
| CTGF | connective tissue growth factor | 14219 | AGCAGCTGGGAGAACTGTGT  GCTGCTTTGGAAGGACTCAC |
| ANKRD1 | ankyrin repeat domain 1 | 107765 | TTGTGAAGGAGCCAGAACCT  CGCCAAGTGTCCTTCTAAGC |
| CCN1 | cellular communication network factor 1 | 16007 | CAAGAAATGCAGCAAGACCA  GGAACCGCATCTTCACAGTT |

| flag-VDR | Forward Primer  Reverse Primer | 5’-3’ GACGAGCTGTACAAGGGATCAATGGAGGCAATGGCGGC  5’-3’ GTCATCCTTGTAGTCGGATCAGATCTCATTGCCAAA |
| --- | --- | --- |
| Sh YAP1 A | Full Hairpin Sequence | TGCTGTTGACAGTGAGCGCCAGGTGATACTATCAACCAAATAGTGAAGCCACAGATGTATTTGGTTGATAGTATCACCTGTTGCCTACTGCCTCGGA |
| Sh YAP1 B | Full Hairpin Sequence | TGCTGTTGACAGTGAGCGCTAGCTCAGATCCTTTCCTTAATAGTGAAGCCACAGATGTATTAAGGAAAGGATCTGAGCTATTGCCTACTGCCTCGGA |

**Supplemental Table 2. Primers for indicated plasmids constructions and shRNA sequences**

| flag-VDR | Forward Primer  Reverse Primer | 5’-3’ GACGAGCTGTACAAGGGATCAATGGAGGCAATGGCGGC  5’-3’ GTCATCCTTGTAGTCGGATCAGATCTCATTGCCAAA |
| --- | --- | --- |
| Sh YAP1 A | Full Hairpin Sequence | TGCTGTTGACAGTGAGCGCCAGGTGATACTATCAACCAAATAGTGAAGCCACAGATGTATTTGGTTGATAGTATCACCTGTTGCCTACTGCCTCGGA |
| Sh YAP1 B | Full Hairpin Sequence | TGCTGTTGACAGTGAGCGCTAGCTCAGATCCTTTCCTTAATAGTGAAGCCACAGATGTATTAAGGAAAGGATCTGAGCTATTGCCTACTGCCTCGGA |
| flag-VDR | Forward Primer  Reverse Primer | 5’-3’ GACGAGCTGTACAAGGGATCAATGGAGGCAATGGCGGC  5’-3’ GTCATCCTTGTAGTCGGATCAGATCTCATTGCCAAA |
| Sh YAP1 A | Full Hairpin Sequence | TGCTGTTGACAGTGAGCGCCAGGTGATACTATCAACCAAATAGTGAAGCCACAGATGTATTTGGTTGATAGTATCACCTGTTGCCTACTGCCTCGGA |
| Sh YAP1 B | Full Hairpin Sequence | TGCTGTTGACAGTGAGCGCTAGCTCAGATCCTTTCCTTAATAGTGAAGCCACAGATGTATTAAGGAAAGGATCTGAGCTATTGCCTACTGCCTCGGA |

Abbreviations

| NLRP3 | NOD-like receptor protein 3 |
| --- | --- |
| BDL | bile-duct ligation |
| YAP1 | yes-associated protein 1 |
| BA | bile acid |
| ASC | apoptosis-associated speck-like protein |
| HSC | hepatic stellate cell |
| VDR | Vitamin D receptor |
| ND | normal diet |
| ALT | alanine aminotransferase |
| AST | aspartate transaminase |
| AKP | alkline phosphatase |
| TBil | total bilirubin |
| TBA | total bile acid |
| FBS | fetal bovine serum |
| LPS | Lipopolysaccharides |
| ATP | adenosine triphosphate |
| LD | Low doses |
| HD | high doses |
| IHC | immunohistochemistry |
| α-SMA | alpha-smooth muscle actin |
| CK-19 | cytokeratin-19 |
| DR | ductular reaction |
| CTGF | connective tissue growth factor |
| ANKRD1 | ankyrin repeat domain 1 |
| CCN1 | cellular communication network factor 1 |
| Co-IP | Co-immunoprecipitation |
| XBP1 | X-box binding protein 1 |


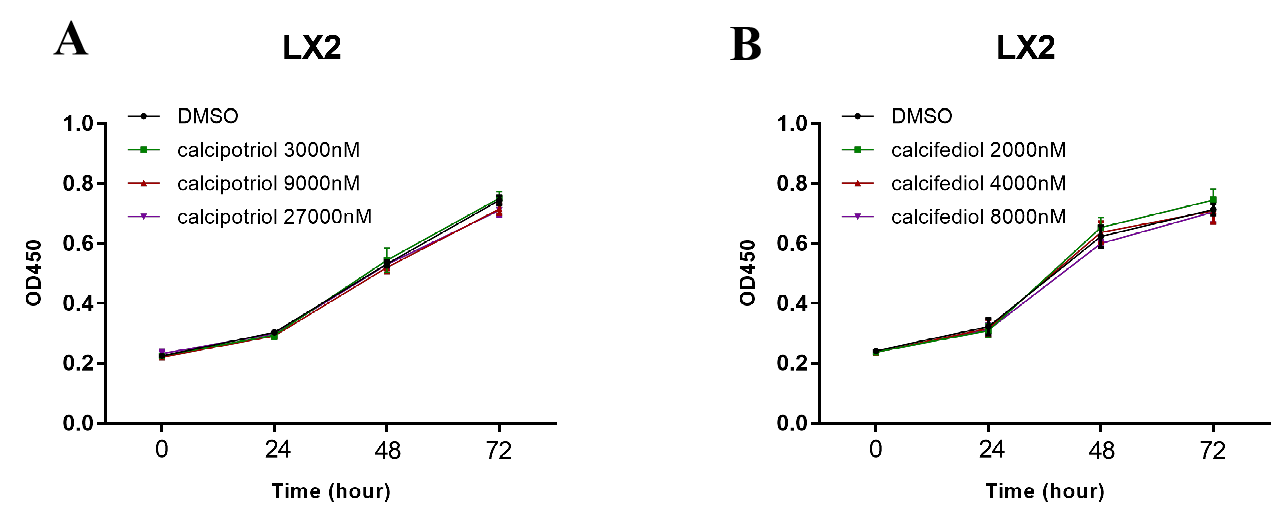


Supplementary Fig. 1. LX2 cell viability was analyzed by CCK-8 assay.  (A) The doses of calcipotriol used in vitro study showed no significant effect on LX2 cell viability. (B) The doses of calcifediol used in vitro study showed no significant effect on LX2 cell viability


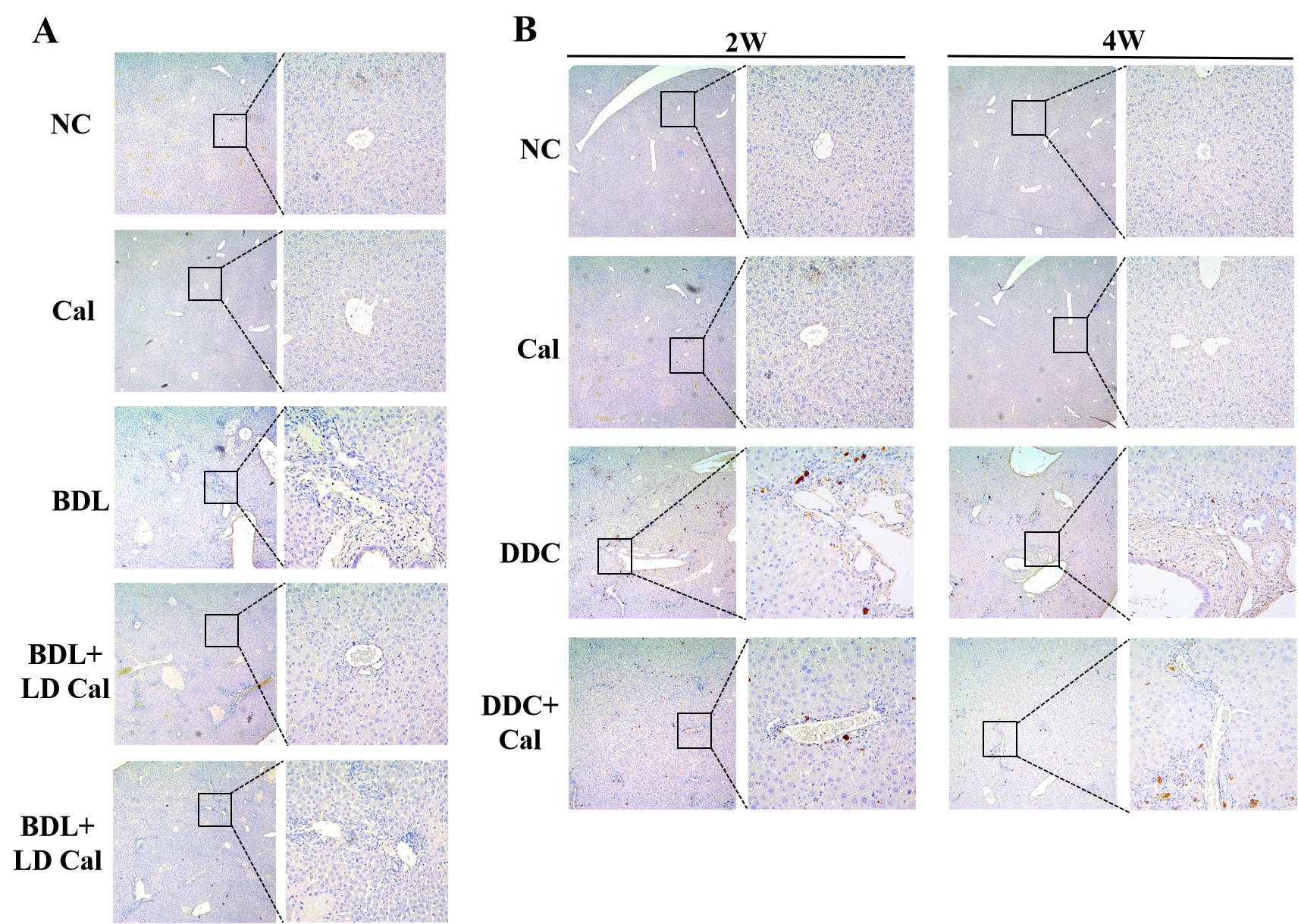


Supplementary Fig. 2. Representative mouse liver samples for Col1A1 (A) IHC staining for of Col1A1 representative mouse liver samples of Sham, Cal, BDL, BDL+LD Cal, and BDL+HD Cal groups. Scale bar = 100 μm. (B) IHC staining for of Col1A1 representative mouse liver samples of ND, Cal, DDC, DDC+Cal at 2 weeks and 4 weeks. Scale bar = 100 μm.


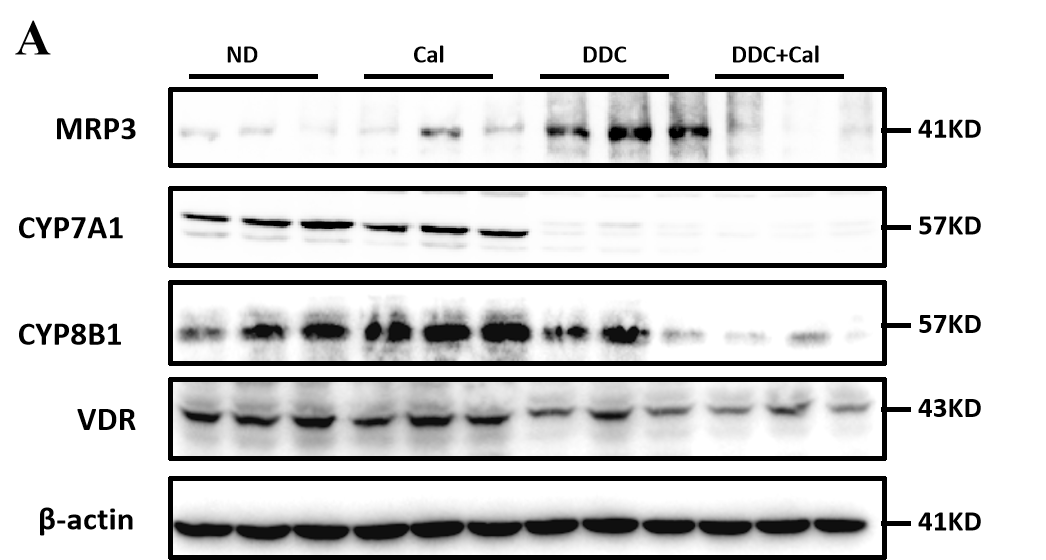


Supplementary Fig. 3. modulates synthesis and transport of BA (A) Protein levels of MRP3, CYP7A1, CYP8B1 and VDR were detected by conducting Western blot analysis and β-actin was used as the control.
